# The Successful Elimination of Sylvatic Rabies Using Oral Vaccination of Foxes in Slovenia

**DOI:** 10.3390/v13030405

**Published:** 2021-03-04

**Authors:** Danijela Černe, Peter Hostnik, Ivan Toplak

**Affiliations:** Institute of Microbiology and Parasitology, Virology Unit, Veterinary Faculty, University of Ljubljana, 1000 Ljubljana, Slovenia; peter.hostnik@vf.uni-lj.si (P.H.); ivan.toplak@vf.uni-lj.si (I.T.)

**Keywords:** rabies virus, Slovenia, oral rabies vaccination (ORV)

## Abstract

Sylvatic rabies was present in Slovenia between 1973 and 2013, with the red fox as the main reservoir of the rabies virus. The first oral rabies vaccination (ORV) control program in foxes started in 1988, using the manual distribution of baits. Significant improvement of fox vaccination was achieved with the aerial distribution of baits, starting in 1995 and successfully finished with the final, fifty-ninth vaccination campaign in 2019. Between 1979 and 2019, a total of 86,471 samples were tested, and 10,975 (12.69%) rabies-positive animals were identified. Within the ORV, two different vaccines were used, containing modified live virus strain Street Alabama Dufferin (SAD) B19 and SAD Bern, while the last ORV campaigns were completed in 2019, with a vaccine containing a genetically modified strain of SPBN GASGAS. Molecular epidemiological studies of 95 rabies-positive samples, originating from red foxes, badgers, cattle, dogs, martens, cats, and horses, revealed a low genetic diversity of circulating strains and high similarity to strains from neighboring countries. During the elimination program, few vaccine-induced rabies cases were detected: three in red foxes and one case in a marten, with no epidemiological relevance. Slovenia has been officially declared a country free of rabies since 2016.

## 1. Introduction

Rabies is a fatal viral disease that affects all warm-blooded mammals, including humans. The disease can be maintained in wild and domestic animals and transmitted to a susceptible host almost exclusively through biting [1]. It is caused by an enveloped RNA virus, of the family *Rhabdoviridae*, genus *Lyssavirus*, and order *Mononegavirales* [2]. Within the *Lyssavirus* genus, 17 species have been described and detected from different parts of the world, with the rabies virus as the most predominant species [3].

Due to the fatal cases and high economic impact on human health systems, numerous effective control measures for rabies as a zoonotic disease have been implemented in affected countries. The disease remains present worldwide, except for in Europe, where most of the countries are currently free of rabies as a result of several decades of running systematic rabies elimination programs. In the past, rabies was continuously detected in the dog population in Europe, and then was progressively eliminated in the 1970s by implementing the mandatory vaccination of dogs and other control measures [4]. At the beginning of the 1940s, sylvatic rabies in Europe began to appear among foxes, and spread progressively from Poland to central and western Europe [5]. These epidemiological changes from urban to sylvatic rabies required a new disease control strategy [6]. The first successful implementation of a rabies elimination program by using oral rabies vaccination (ORV) of foxes started in Switzerland in 1978 [7]; starting in 1984, the same approach was adopted in several European countries, resulting in rapid decreases of the number of reported rabies cases. In 2021, rabies has been eliminated from the western and central parts of Europe, but remains present in some countries of the eastern part of Europe, such as Lithuania, Poland, Hungary, and Romania [8]. Recurrences of rabies in Italy [9], Greece [10], and Slovakia showed the need for continuous surveillance of the epidemiological situation within the European Union (EU) in wildlife and domestic animals [11].

Slovenia is one of the smallest countries within the EU. It is in central Europe, and has national borders with Italy, Austria, Hungary, and Croatia. Slovenia covers 20,271 km^2^ of land and has a population of 2.095 million people. With 58.3% of the territory covered by forests, it is the third most forested country in Europe. In Slovenia, urban and sylvatic types of rabies have presented a major health threat for several decades. In 1947, the mandatory rabies vaccination of dogs was introduced to decrease and finally prevent urban rabies cases [12]. After the implementation of the systematic mandatory vaccination program of dogs, run annually by veterinarians, and with the increasing of awareness, the incidence of urban rabies was reduced, and the last rabid dog was recorded in 1954. The last officially recorded case of human rabies in Slovenia was recorded in 1950 [13]. Later, no further rabies-fatal human cases were recorded, which is also the result of close cooperation of the veterinary sector and public health institutions. The hunting bag of red foxes each year consisted of about 10,000 animals, representing the most predominant carnivore species in Slovenia [14]. The first positive case of sylvatic rabies in Slovenia was recorded in 1973 in the northeastern part of the country (the Prekmurje region), near the border with Hungary [12], and the red fox was identified from the beginning of epizooty as the main reservoir [15]. In 1979, a second independent wave of sylvatic rabies was detected in Slovenia in two different regions on the national border with Austria (the Koroška and Gorenjska regions). In the following years, the number of detected rabies-positive animals in the country increased rapidly. The implementation of a national ORV program led to a decreased number of detected positive wild animals.

During the almost four decades of elimination programs, close cooperation between the Administration of the Republic of Slovenia for Food Safety, Veterinary and Plant Protection (AFSVPP), the National Veterinary Institute (NVI), hunting organizations, and veterinary organizations was established. The AFSVPP was responsible for financial funding, ORV organization, data collection, statistical analyses, and rabies public awareness. Hunter organizations were included in the manual distribution of rabies vaccine baits in the first ORV trials, and were contracted by the AFSVPP to shoot a set number of foxes. Veterinary organizations were included in organization of quarantine, sample reception, data recording, and sending samples to the laboratory. The NVI was responsible for the transport of samples and pathological sections, while its virology unit was responsible for laboratory rabies diagnostics, data collection, reporting to the AFSVPP, and expert scientific support. In 1970, the fluorescent antibody test (FAT) for laboratory rabies detection was introduced as a routine laboratory test, and was accredited under EN ISO/IEC 17025 in 2006. Methods for vaccination efficacy evaluation by the detection of antibodies in blood samples and oxytetracycline-specific fluorescence in the tooth and alveolar bone tissue of captured foxes were implemented in 1995. In 2010, the first laboratory molecular methods were introduced to enable the first molecular characterization of the rabies virus strains and molecular epidemiology studies in Slovenia [16]. Since 2008, the virology unit of the NVI has been the National Reference Laboratory (NRL) for rabies, and is included in the network of the European Reference Laboratory (EURL) for rabies led by the ANSES, France. Since 1996, the virology unit has been participating annually in the international proficiency testing scheme organized by the EURL or other laboratories. In 2021, the virology unit of the NVI in Ljubljana is performing a total of 11 different methods for complete rabies diagnostics. The implementation and continuous improvement of laboratory diagnostic capacities for the detection of the rabies virus and monitoring the effectiveness of mandatory vaccination programs of dogs, the ORV program, post-exposure prophylactic measures, and educational campaigns have all contributed to Slovenia finally being declared a rabies-free country.

In the neighboring country of Croatia, the first wave of sylvatic rabies was recorded in 1977 [17]. The first attempt of the ORV of foxes was in the 1990s at a small scale, along the Istria peninsula and borders of Slovenia. In 2011, the countrywide ORV program began, supported by the European Commission, and the number of rabies-positive cases rapidly decreased until 2014, when the last case was confirmed [18].

At the end of the 1930s, the neighboring country of Hungary was the first European country to succeed in controlling urban rabies via the compulsory preventive rabies vaccination of dogs and the implementation of legislation concerning keeping dogs [19]. Sylvatic rabies was recorded for the first time in 1954 [20]. ORV campaigns started in 1992, and the incidence of rabies cases decreased [21]. In 2013, rabies re-emerged, and protective ORV within a radius of 50 km from the outbreak center was performed. The last recorded case of rabies was detected in 2017 in the vaccination area along the border with Slovakia [22].

The first sylvatic rabies case in Austria was recorded in 1966 [19]. The ORV program started in 1986, resulting in a radical reduction of positive cases. In 2002, 24 cases were detected during one outbreak, located near the Slovenian border; the outbreak was successfully controlled with emergency ORV [23]. The last reported case of rabies in Austria was in 2006, but the border vaccination zone was maintained until 2012, due to individual cases of rabies in Slovenia and a re-occurrence in Italy [24].

The neighboring country of Italy eliminated urban rabies in 1973, but sporadic occurrences in wild animals were reported in the late 1960s onward [25]. Between 1997 and 2008, Italy was declared free of rabies [9]. On 17 October 2008, the National Reference Centre identified a rabid fox, located near the border with Slovenia, and an emergency ORV program was organized in the infected area [26]. ORV in Italy continued until 2014 to protect the population of foxes on the border with Slovenia and to prevent rabies re-introduction [9].

The objective of this study was to present the collected results and experiences of four decades of control and elimination of sylvatic rabies in Slovenia within the national rabies elimination program. The efficiency of different ORV models, the incidence of rabies, bait consumption, and immunization rates in Slovenia were reviewed.

## 2. Materials and Methods

The first ORV of foxes in Slovenia started in 1988 in the northwestern part of Slovenia, along the Austrian and Italian borders [15,27]. In the beginning, the ORV was performed manually by hunters, with vaccine baits containing a modified live virus SAD B19 (IDT Biologika GmBh, Dessau-Rosslau, Germany). Between 1988 and 1993, two ORV campaigns were performed each year, from April to May and from October to November. The aim of the first ORV was to eliminate sylvatic rabies, first in the western part of Slovenia, and then in the central and finally the eastern parts of the country. Manual bait distribution was performed according to the net system, with an achieved density of 16 up to 20 baits per km^2^, covering from 4000 to 11,000 km^2^ of land area in a total of nine campaigns. From 1988 to 1993, between 64,000 and 220,000 baits were distributed yearly.

**Figure 1:**
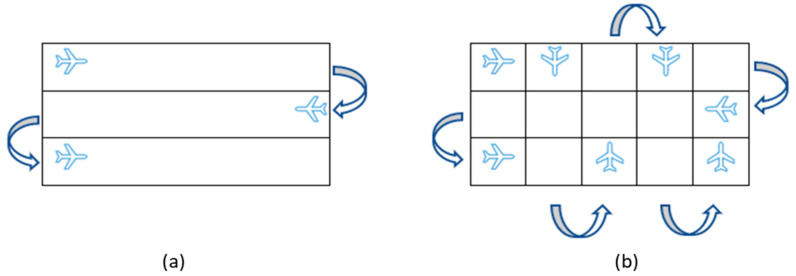
The pathways of aerial bait distribution in parallel flights (**a**) and in crisscross flights (**b**).

A second ORV model started in 1995, and all baits during each campaign were distributed by aircraft. Depending on the terrain, the aircraft flew at an altitude between 200 and 500 m, at a speed of 100–150 km/h. Flight paths were in parallel lines 1000 m apart (Figure 1a). After 2003, parallel flights were replaced with crisscross flights: flight paths in parallel lines followed flights in the same area in a perpendicular direction to the first flight lines (Figure 1b). For navigation, orientation, and data recording for the exact location and time of baits dropping, the aircraft used the global positioning system (GPS) and were equipped with the FIC^®^ computer program [15]. No baits were dropped in urban areas, or over rivers, lakes, or in high mountains. A cold chain with a temperature below –20 °C was provided and recorded for each batch of baits until bait distribution.

In the second ORV model, three different vaccine baits were used. Vaccine strain SAD B19 (IDT Biologika GmBh, Dessau-Rosslau, Germany) was used between 1995 and 2018. Between 1998 and 2001, strain SAD Bern (Bioveta, Ivanovice na Hané, Czech Republic) was used only on the western part of the vaccinated area of Slovenia. From autumn 2018 to autumn 2019, only vaccines containing the genetically modified strain SPBN GASGAS (IDT Biologika GmBh, Dessau-Rosslau, Germany) were used. Together with all three vaccine strains, oxytetracycline (150 mg/bait) was used as a marker, causing specific fluorescence in the tooth and alveolar bone tissue after bait uptake. To control the rate of bait consumption, oxytetracycline-specific fluorescence was inspected by using a fluorescent microscope. The second ORV model was continuously performed between 1995 and 2019, with a total of 50 campaigns. Each year, baits were distributed from April to June and from October to November. Around 920,000 baits were distributed each year to achieve a density between 21 and 25 baits per km^2^, covering the entire territory of Slovenia. In the previous three years (between 2017 and 2019), ORV was performed only along the border with Croatia, covering a protective zone of 15,000 km^2^.

Evaluation of the ORV elimination program was performed each year by calculating the rabies incidence, evaluation of bait consumption, immunization rate, and the genetic characterization of representative strains circulating in Slovenia. The rabies incidence in Slovenia was determined on the laboratory testing results of wild and domestic animals. Within active and passive surveillance, brain tissue samples were conducted to direct rabies diagnosis by FAT assay, all performed in accordance with WHO standards [28], at the NVI virology unit in Ljubljana. From 2010 to 2020, all inconclusive or positive results by FAT were also confirmed using RT-PCR methods.

Since 2005, the evaluation of fox bait consumption was assessed via the detection of the marker oxytetracycline, with specific fluorescence in the tooth and alveolar bone of randomly selected foxes. Random selection of samples was calculated by the AFSVPP to cover the vaccination area and according to the EURL for rabies (ANSES, France) recommendations [29]. The recommended sample size for Slovenia was 808 foxes annually. To evaluate the presence of oxytetracycline-specific fluorescence with a fluorescence microscope, 200–300 µm-thick tooth sections or lower jaw periodontal bone sections were prepared.

Since 2005, rabies immunity was determined using antibody detection in samples of thoracic or abdominal fluids from foxes collected throughout each year using the commercial indirect ELISA test Bio-Rad (Platelia Rabies II, Marnes-la-Coquette, France). In 2013, a new ELISA kit with higher sensitivity was implemented (BioPro Rabies ELISA Ab kit, Prague, Czech Republic) [30]. The presence of antibodies found using ELISA was evaluated according to the manufacturer’s instructions, using a cutoff value of 0.5 EU/mL for the ELISA test Bio-Rad (Platelia Rabies II, Marnes-la-Coquette, France) and ≥40% percentage of blocking for BioPro Rabies ELISA (Prague, Czech Republic).

Between 1994 and 2018, 2633 FAT-positive samples were detected, and 159 were archived at the NVI virology unit (Supplementary Materials Table S1). For the genetic characterization of strains circulating in Slovenia, a total of 95 FAT-positive samples were selected according to the availability of the original archive brain samples, year of collection, geographical location, and to cover different animal species. Ninety-five samples included in further genetic analysis originated from red foxes (*n* = 83), badgers (*n* = 3), cattle (*n* = 3), dogs (*n* = 2), martens (*n* = 2), a cat (*n* = 1), and a horse (*n* = 1) [15]. Total viral RNA was extracted from the selected brain samples using the QIAamp^®^ Viral RNA Mini Kit (Qiagen, Düsseldorf, Germany) according to the manufacturer’s instructions. Reverse transcription (RT) with polymerase chain reaction (PCR) was performed in one tube using a One-Step RT-PCR Kit (Qiagen, Düsseldorf, Germany) with the primer sets N7 (5′-ATG TAA CAC CTC TAC AAT G-3′) and N4 (5′-GTC TGA TGA TTG GAA CT-3′) to amplify 1313 bp long products of nucleoprotein gene [31]. Partial N gene nucleotide sequences of selected positive samples were assembled using the DNASTAR (version 5.05) program and compared to the known sequences of the N gene in the GenBank database. A multiple alignment of the nucleotide and protein coding sequences was performed using Clustal W and neighbor-joining criteria. A phylogenetic tree based on a 1092-nucleotide sequence of the N gene was constructed using the Phylip program [32] and the maximum likelihood algorithm with strain PV (GU992322) as an outgroup. Bootstrap values were obtained for 1000 datasets.

## 3. Results

### 3.1. The Incidence of Rabies in Slovenia

**Figure 2:**
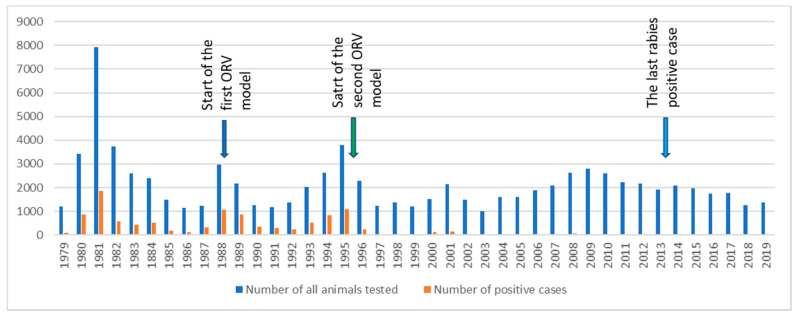
The number of tested animals (blue) and number of FAT-positive animals (orange) between 1979 and 2019 in Slovenia. The arrows show years with important dates: 1988, start of the first ORV model; 1995, start of the second ORV model; and 2013, the last sylvatic rabies case detected in Slovenia.

Sylvatic rabies was firstly detected in a red fox on 3 September 1973 in the eastern part of Slovenia and then locally spread, mainly among red foxes in the Prekmurje region. The disease was localized with a natural border, the River Mura, which was not crossed until 1979. In 1979, the second wave of sylvatic rabies was identified at two different locations in the northern part of Slovenia (at borders with Austria), from which the disease then rapidly spread throughout the country. Systematic data collection on the incidence of rabies in Slovenia started in 1979. Three peaks with the highest number of detected positive cases were identified during the epizooty: the first in 1981 with 1851 (23.39%) rabies-positive cases, the second in 1988 with 1067 (36.01%) positive cases, and the third in 1995 with 1089 (28.76%) positive cases, while the last rabies-positive animal was detected in January 2013 (Figure 2). From 1979 to 2019, a total of 86,471 animals were tested, from which 66,858 were red foxes, 6050 cats, 3972 dogs, 3158 roe deer, 1917 martens, 1772 badgers, 677 cattle, 78 wild boars, 34 horses, and 1955 other animals. In this period, of the 86,471 tested samples, 10,975 (12.69%) were detected as rabies positive. The majority of positive cases were detected among red foxes, with 9967 (90.81%) positive, followed by badgers with 241 (2.19%), roe deer with 215 (1.96%), cats with 207 (1.89%), dogs with 119 (1.08%), martens with 118 (1.07%), cattle with 45 (0.41%), sheep with 7 (0.06%), horses with 5 (0.04%), wild boars with 3 (0.03%), and rabbit with 1 (0.01%), while 47 (0.43%) positive samples belonged to other wild animals (red deer, skunk, squirrel, rats, hedgehog, wolves, lynx, mouflon, and chamois) (Table S2). The most frequently infected species within domestic animals was cats, followed by dogs, cattle, sheep, horses, and rabbits.

When comparing the first and second ORV models, great differences among the rabies incidence can be observed (Table S2). Within the first ORV model, the rabies incidence ranged between 38.84% in 1989 to 17.44% in 1992. In the second ORV model, the rabies incidence rapidly decreased from 28.76% in 1995 to 0.00% in 2015.

**Figure 3:**
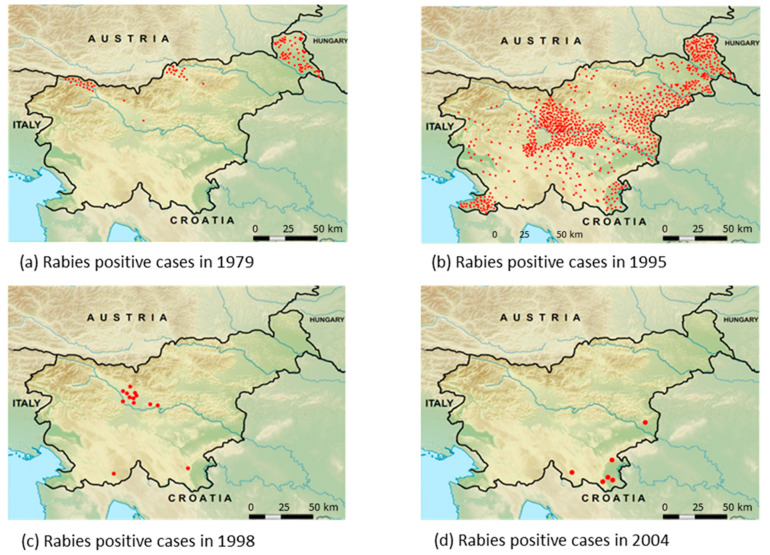
The detected rabies-positive cases by FAT in Slovenia in 1979 (**a**), 1995 (**b**), 1998 (**c**), and 2004 (**d**).

In 1979, rabies was present only in the northern and eastern parts of Slovenia from where it spread; in 1995, positive cases were detected in all regions of the country (Figure 3a,b). The effectiveness of the second ORV model was proved in 1998 when the disease was detected only in the central part of the country (near the international airport) and with a few cases in the area near the Croatian border (Figure 3c). In 2003, parallel flights were replaced with crisscross flights, which resulted in the reduction of positive cases from the central part of Slovenia. Since 2004, rabies-positive cases were detected only near the border with Croatia (Figure 3d).

### 3.2. Bait Consumption and Immunization Rate

**Figure 4:**
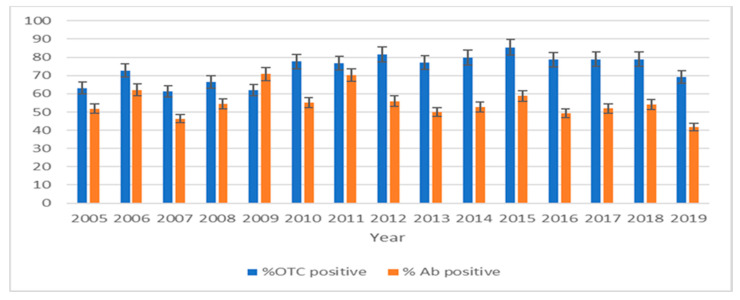
The percentage of oxytetracycline-positive (% OTC positive) and the percentage of rabies antibody-positive foxes (% Ab positive) between 2005 and 2019 in Slovenia with presented 95% confidence limits.

Within the evaluation of the effectiveness of the ORV program, the percentages of oxytetracycline-specific fluorescence-positive foxes were determined between 2005 and 2019. A total of 13,111 samples were tested, with an average of 72.75% (CI: 69.11–76.39%) detected oxytetracycline-specific fluorescence positive samples, and with 95% confidence intervals. The lowest percentage (61.19%; CI: 58.13–64.22%) of oxytetracycline-specific fluorescence-positive samples was detected in 2007, and the highest (85.51%; CI: 81.23–89.79%) in 2015 (Figure 4).

In the same period, the immunization rate was determined using commercial indirect ELISA for the specific detection of antibodies in fox serum samples. Between 2005 and 2019, a total of 8280 samples were tested by ELISA, with an average of 54.20% (CI: 51.49–56.91%) positive samples. The detected percentage of rabies antibody-positive samples ranged from the lowest, 41.72% (CI: 39.66–43.84%) in 2019 to the highest, 70.78% (CI: 67.24–74.32%) in 2009 (Figure 4).

### 3.3. Genetic Characterization of Rabies-Positive Samples

**Figure 5:**
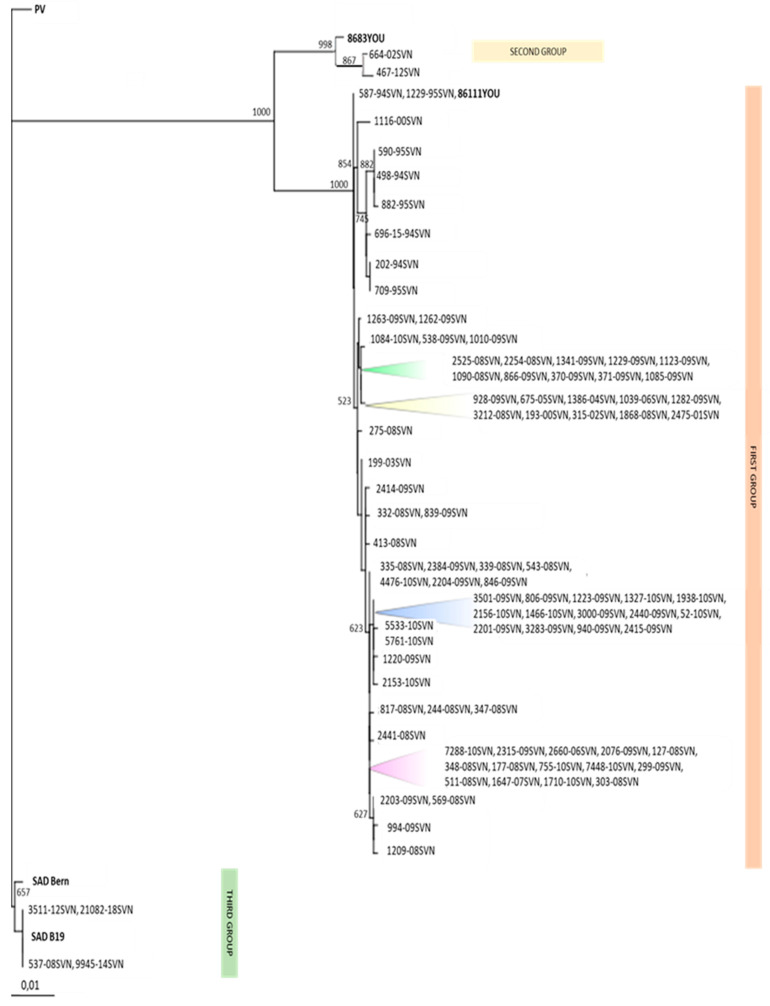
Phylogenetic tree based on a 1092-nucleotide sequence of the N gene of rabies virus was constructed with the Phylip program by using the maximum likelihood algorithm with strain PV (GU992322) as an outgroup. Bootstrap values were obtained for 1000 datasets. Out of 95 Slovenian sequences, 89 clustered into the first group closely related to the sequences from WE, two clustered into the second group closely related to the sequences from EE, and four clustered into the third SAD B19 vaccine-associated group.

A total of 95 positive samples detected in Slovenia between 1994 and 2018 were genetically characterized by sequencing. All characterized strains from Slovenia belonged to the classic rabies virus (RABV). The genetic comparison of rabies strains originated from different animal species revealed that the red fox was the main reservoir of the rabies virus in Slovenia. The phylogenetic comparison of 1092 nucleotide sequences of the N gene for strains detected in Slovenia showed 91.8–100% nucleotide homology to each other and 94.6–100% nucleotide homology to the other European rabies isolates from the GenBank database (9202ALL (U42701), 9244FRA (U42607), 86107YOU (U42703), 8653YOU (U42704)). Phylogenetic comparison of 95 nucleotide sequences revealed that the Slovenian strains were clustered into three genetically related groups (Figure 5). According to a previously established classification of European rabies nucleotide sequences [31], 89 Slovenian sequences originating from red foxes, badgers, cattle, dogs, marten, a cat, and a horse were clustered into the first Western Europe (WE) group with 99.0–100% nucleotide identity between them; two were clustered into the second Eastern Europe (EE) group with 98.6% identity between two strains detected in red foxes; and four originating from red foxes and martens were clustered into the third SAD B19 vaccine-associated group, all with 100% nucleotide identity. Rabies strains from the first WE group and the second EE group revealed 95.9–96.4% nucleotide homology; the first WE group and the third SAD B19 vaccine-associated group shared 91.8–92.3% nucleotide homology. The second EE group and the third SAD B19 vaccine-associated group had 92.5% nucleotide homology.

The genetic comparison of nucleotide identity with the strain from Italy red fox/08RS-1981/Udine/2008 (JF424484) [33] revealed 100% identity to Slovenian strain 275-08SVN (HM852154). The comparison of rabies virus strain 4765/14 (KX929158) from Croatia [34] revealed 100% identity to stain 75510SVN (HM852158) from Slovenia. When comparing Slovenian strains to strain RV1538 (JF973836) from Austria [35], the highest percentage of identity (99.82%) was detected with strain 199-03SVN (HM852155).

Among the 95 characterized rabies strains in Slovenia, four vaccine-induced rabies cases (4.21%) were identified: three in red foxes and one in a marten. The comparison of 1092 nucleotide sequences of the N gene of four vaccine-induced cases showed 100% homology to the reference SAD B19 strain (EF206709). The first vaccine-positive case 537-08SVN was detected during routine FAT testing of a less than one-year-old fox, which was found dead in a backyard in the municipality of Šentjur in February 2008, three months after a vaccination campaign. The second vaccine-positive case was identified in a less than one-year-old red fox 3511-12SVN in the municipality of Tolmin in May 2012, during a vaccination campaign. The third vaccine-positive case was detected in a marten 9945-14SVN in the municipality of Ljubljana in November 2014 during a vaccination campaign, and the last case in a two-month-old red fox 21082-18SVN, which was found dead in a backyard in the municipality of Ljubljana in June 2018, one month after a vaccination campaign.

All detected vaccine-induced rabies viruses were analyzed by FAT, cell culture virus isolation, RT-PCR, and sequencing (Table 1). Vaccine-induced rabies virus 3511-12SVN (KC522613) was first detected by FAT from a red fox in 2012, and an infective vaccine virus was isolated from brain samples and salivary glands. The fox showed unusual behavior and was shot by a hunter. Detailed molecular analysis of a 4351-nucleotide long fragment between the N ad G genes (KC522613) of vaccine-induced isolate 3511-12SVN and vaccine strain SAD B19 showed two nucleotide variations at position G1335A of the N gene and A3114G of the M gene noncoding region. Nucleotide variation in the N gene resulted in one amino acid exchange from Arg to Gln at amino acid position 422 [36].

**Figure 6:**
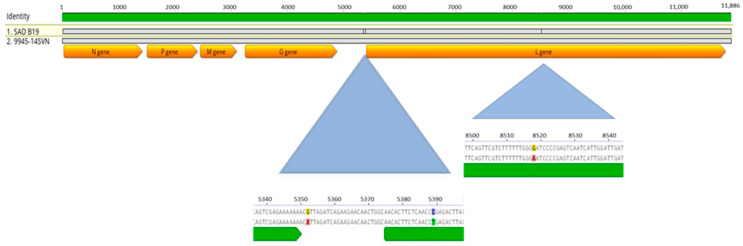
Complete genome of vaccine-induced rabies virus 9945-14SVN detected in the brain tissue of an FAT-positive marten. Nucleotide changes were found at positions A5352G, T5389C, and A8518G, according to vaccine virus SAD B19.

In 2014, the third vaccine-induced rabies virus 9945-14SVN was detected in a marten. The animal was found during the day in a village and showed uncoordinated movement and aggression. The complete genome sequence of 9945-14SVN was determined with the NGS, revealing only three nucleotide changes in 11,886 determined nucleotides in comparison with vaccine strain SAD B19. The nucleotide variations were found on the following genome positions: A5352G, T5389C, and A8518G (Figure 6). The nucleotide variation of the L gene at position 5389 resulted in Arg amino acid creation instead of termination.

## 4. Discussion

In Slovenia, the urban and sylvatic types of rabies were widespread over the last century, and presented a major threat to human health. In 1947, the mandatory vaccination of dogs was implemented throughout Slovenia; as a result, the incidence of dog rabies was sharply reduced, and urban rabies was eliminated in 1954 [12]. The population of domestic cats has never been mandatorily vaccinated in Slovenia, since cats are exclusively territorial animals, and the potential transmission to the healthy population was, according to the veterinary service, very low. The sylvatic type of rabies was first detected in 1973 in the eastern part of Slovenia. The second incursion of sylvatic rabies in Slovenia was detected in 1979 in the northern part of the country, in two separate geographic areas (the Koroška and Gorenjska regions), from which the disease spread throughout the country. The red fox was identified as the main reservoir species in Slovenia, with the highest percentage of rabies-positive results (90.81%). Although badgers may be reservoirs in some geographic areas [37], the identification of a very low percentage of positive samples (0.02%) in the population of badgers suggests that the population is too small to become a reservoir in Slovenia. A potential rabies reservoir species in Europe is the golden jackal [38], whose population is expanding and also present in Slovenia [39]. If the population densities continue to increase, jackals could maintain the independent rabies transmission cycle [38].

The first ORV model with manual bait distribution, running in Slovenia between 1988 and 1993, did not significantly decrease the sylvatic rabies incidence due to high human impact and insufficient and instable financial resources during the first five years of the ORV campaign. Vaccination was implemented only in a small geographic area, and the constant detection of rabies-positive red foxes in infected areas was present. Although the laboratory results showed a reduction of rabies-positive cases after each ORV campaign, this was not effective in eliminating rabies in Slovenia [40]. The second ORV model with aerial bait distribution was implemented in Slovenia in 1995, and the veterinary services (AFSVPP) increased the budget for financial funding of ORV. Between 1995 and 2017, ORV was performed each year in the entire territory of Slovenia. The great success of the second ORV has resulted in a decreasing number of positive cases. After 2003, a low number of positive cases were detected only in the central region of the country, with an additional few cases in the area next to the border with Croatia, as a result of natural cross-border transmission. The adoption of the second ORV model, in which parallel flights were replaced by crisscross flights, resulted in rabies elimination from the central part of the country. Crisscross flights proved to be more effective with better bait distribution and coverage in valleys and hills [15]. With the decreasing number of positive animals and the favorable epizootiological situation in Hungary, Austria, and Italy, the ORV area was gradually reduced in 2017. Since 2004, the majority of positive cases in Slovenia were detected in the wider area along the border with Croatia, attributable to the epidemiological situation in that country, where ORV was implemented in 2011 [18]. ORV in Croatia also resulted in a decreasing number of detected positive rabies cases, and epizooty of sylvatic rabies in Slovenia was finished with the last fox rabies-positive sample in 2013. According to the favorable epidemiological situation in Slovenia and the neighboring area, the final ORV was performed between 2017 and 2019, only partially covering a zone of 15,000 km^2^ along the border with Croatia.

In Slovenia, the ORV of foxes ran for a total of 31 years (59 campaigns of ORV were performed), until the elimination of sylvatic rabies in the red fox was completed, while in some countries, sylvatic rabies was successfully eliminated by ORV in only three years [41]. The time to eliminate rabies by ORV depends on several factors, and is strongly related to the geographic area of each country, the epidemiological situation, and cross-border cooperation in ORV campaigns. Although the coordinated ORV campaigns with the neighboring countries of Austria, Hungary, and Italy started in Slovenia with the first ORV, the start of ORV campaigns in Croatia had a great final impact on successful rabies elimination in Slovenia.

Within the first ORV model, the main obstacles to success were economic and social factors. Hunters were working voluntarily, which led to decreasing motivation after a few ORV campaigns. In the second ORV model, the AFSVPP provided stable and continuous financial funding, which was critical to the success of each year’s ORV campaign and laboratory testing for the detection of incidence of rabies, bait consumption, and immunization rates. Each year, vaccine selection was strongly dependent on price and vaccine availability. The vaccine used in ORV campaigns influences the duration of rabies elimination by ORV [42]. In most of the ORV campaigns in Slovenia, the vaccine containing SAD B19 was used. Some previous observations demonstrated faster and more durable decreases in the rabies incidence when using SAG and V-RG vaccines in comparison with SAD B19 [43], which was one of the main reasons that Slovenia finished ORV with a vaccine containing genetically modified strain SPBN GASGAS (IDT Biologika GmBh, Dessau-Rosslau, Germany).

In the scope of the evaluation of ORV in Slovenia, bait consumption and immunization rates showed the expected results after each campaign. According to the recommendations of international organizations about the sample size for bait consumption and immunization rate evaluation [29,44], the sample size for Slovenia was 808 foxes annually. This recommendation was strictly followed between 2005 and 2010, and in 2013 and 2016. In the other years, the numbers of tested foxes were lower, but in all years was above 617. The observed discrepancy between the percentage of bait consumption and immunization rate was similar in other European countries due to low-quality serum samples, because the duration and conditions of carcass storage have a significant impact on sample quality [45]. For immunization rate evaluation, different techniques can be used. The most commonly used techniques are ELISA and virus neutralization testing [46], for which low-quality serum samples could react with false positives [45] or cause cytotoxic effects [47]. When comparing the percentage of foxes with bait consumption and antibody detection, better results can be observed when a smaller number of foxes is tested and where more samples originated from animals older than three years, which are more likely to consume the baits [48]. A higher degree of result comparability could be assessed in Slovenia when using a filter paper blood sampling procedure [30].

Phylogenetic comparison of 95 selected rabies positive samples revealed that Slovenian sequences formed three genetically related groups. Some identified strains from the WE group in Slovenia revealed almost 100% nucleotide identity, and were also described in Austrian, Croatian, and Italian positive samples [18,33]. These data explain the natural dynamics of circulating rabies strains in the same geographic area and the identification of genetically closely related strains in neighboring countries. Within the SAD B19 vaccine-associated group, four rabies cases were identified in Slovenia. No nucleotide changes were observed in samples 3511-12SVN and 9945-14SVN in the G protein gene, which is responsible for rabies virulence [49,50,51], also excluding the recombination between wild type and vaccine strain SAD B19. The occurrence of the rabies vaccine virus in the brain tissue of four samples from Slovenia may be the result of the residual pathogenicity of the SAD B19 vaccine strain for rodents. Vaccine-induced cases were also reported from Austria and Germany, suggesting that the most likely explanation for these cases is reversion of virulence or residual pathogenicity of the vaccine virus [24]. None of vaccine-associated cases of rabies resulted in outbreaks, confirming that Slovenian vaccine-associated cases have no epidemiological relevance. During the final ORV, performed between 2018 and 2019, when a modified strain of SPBN GASGAS was used in the vaccine, no new vaccine-induced cases were detected in Slovenia.

Good cooperation between veterinary and human health services in Slovenia resulted in the fact that no human rabies case in Slovenia has been recorded in the last 70 years. In Slovenia, 64% of post-exposure vaccinations were performed due to bites caused by a dog of an unknown owner [52]. The successful elimination of sylvatic rabies in Slovenia also decreased the risk of rabies in the human population to a minimum. The increasing migration of people and importations of potentially rabid animals still represent a sustainable risk for re-introducing rabies into the county; therefore, preventive measures, including the promotion of awareness of the danger of rabies, must be continued.

All accepted biosecurity measures in Slovenia, especially the implementation of ORV, enabled the country to be declared rabies-free in 2016 in accordance with WHO requirements [53]. This success confirmed that the ORV program was one of the most important methods for rabies elimination. According to valuable data and experiences from neighboring countries (Hungary, Austria, and Italy), it is crucial to maintain wildlife rabies monitoring and a high level of awareness of the disease in Slovenia to test and report all suspected cases immediately and implement preventive measures, including emergency ORV in case of the re-emergence of wildlife rabies.

## Figures and Tables

**Table 1:** Laboratory results of four vaccine-induced rabies cases from Slovenia.

Name of Vaccine-Induced Rabies Virus	Animal Species	Detection Period	FAT	Cell Culture Virus Isolation	RT-PCR	Sequencing	NGS
537-08SVN	red fox	February 2008	positive	positive	positive	1092 nt of N gene	nd
3511-12SVN	red fox	May 2012	positive	positive	positive	4351 nt of N-G gene	nd
9945-14SVN	marten	November 2014	positive	positive	positive	1092 nt of N gene	11.886 nt
21082-18SVN	red fox	June 2018	positive	positive	positive	1092 nt of N gene	nd

## Data Availability

All data that support the findings of this study are included within the article and Supplementary Materials.

## Supplementary Materials

The following are available online at https://www.mdpi.com/1999-4915/13/3/405/s1: Table S1: Archive collection of rabies-positive samples at the NVI virology unit, Ljubljana, Slovenia. Table S2: The presentation of detected rabies-positive samples in domestic and wild animal species; data collected between 1979 and 2019 (laboratory archive data from the NVI virology unit, Ljubljana, Slovenia).
