# Supplementary material for: The Successful Elimination of Sylvatic Rabies Using Oral Vaccination of Foxes in Slovenia
Source: Viruses. 2021 Mar 4;13(3):405. doi: 10.3390/v13030405 (PMC8001208; doi:10.3390/v13030405)
Supplement: Supplementary file 1 [file viruses-13-00405-s001.pdf]

viruses MDPI

Table S1. Archive collection of rabies positive samples at the NVI, Virology Unit.

Sample name	Year	Location	Animal species	Genetic characterization (accession number)
202	1994	Jesenice	Cow	Yes (HM852150)
498	1994	Ribnica	Fox	Yes (HM852146)
587	1994	Sežana	Marten	Yes (HM852152)
696-15	1994	Ilirska Bistrica	Fox	Yes (HM852149)
355	1994	Sežana	Fox	No
536	1994	Murska Sobota	Fox	No
477	1994	Krško	Fox	No
579	1994	Nova gorica	Fox	No
34	1994	Murska Sobota	Fox	No
1386	1994	Ribnica	Fox	No
882	1995	Cerknica	Cat	Yes (HM852148)
1478	1995	Domžale	Fox	No
1229	1995	Krško	Fox	Yes (HM852153)
709	1995	Kamnik	Dog	Yes
1453	1995	Tolmin	Fox	No
1376	1995	Novo Mesto	Fox	No
1439	1995	Litija	Fox	No
762	1995	Šmarje	Fox	No
837	1995	Koper	Fox	No
1422	1995	Brežice	Fox	No
1447	1995	Domžale	Fox	No
1089	1995	Šentjur	Fox	No
590	1996	Litija	Fox	Yes (HM852147)
1332	1996	Žalec	Fox	No
540	1996	Ptuj	Fox	No
704	1997	Kamnik	Cat	No
923	1999	Črnomelj	Cat	No
2045	1999	Črnomelj	Fox	No
1116	2000	Novo Mesto	Badger	Yes (HM852151)
193	2000	Litija	Fox	Yes
283	2000	Trebnje	Fox	No
1254-205	2000	Brezovica	Fox	No
876	2000	Semič	Fox	No
660	2000	Škocijan	Fox	No
718	2001	Ravne na koroškem	Cow	No
316	2001	Radovljica	Dog	No
1496	2001	Grosuplje	Dog	No
1516	2001	Majšperk	Cat	No
1807	2001	Ribnica	Dog	No
1795-69	2001	Podlehnik	Fox	No
2475	2001	Koper	Fox	Yes
1555	2001	Litija	Fox	No
1403	2001	Novo Mesto	Fox	No
416	2001	Litija	Fox	No
1558	2001	Litija	Fox	No
2097	2001	Kranj	Fox	No
854	2002	Šoštanj	Dog	No
541	2002	Radovljica	Fox	No
315	2002	Laško	Fox	Yes (HM852164)
664	2002	Videm pri Ptuju	Fox	Yes (HM852145)
1693	2002	Brežice	Fox	No
1945	2002	Brežice	Fox	No
2049	2002	Brežice	Fox	No
255	2002	Videm pri Ptuju	Fox	No
1949	2002	Škofja Loka	Fox	No
517	2002	Škofja Loka	Fox	No
1155	2002	Šmarje pri Jelšah	Fox	No
5174	2002	Videm pri Ptuju	Fox	No
199	2003	Brežice	Fox	Yes (HM852155)
317	2003	Videm pri Ptuju	Fox	No

2337	2003	Vrhnika	Fox	No
2159	2003	Kočevje	Fox	No
2475	2003	Kočevje	Fox	No
514	2003	Ljubljana	Fox	No
404	2003	Brežice	Fox	No
1386	2004	Ilirska Bistrica	Fox	Yes (HM852165)
314	2004	Ilirska Bistrica	Fox	No
820	2004	Semič	Fox	No
675	2005	Polzela	Fox	Yes
305	2005	Žalec	Fox	No
1039	2006	Črnomelj	Fox	Yes
1647	2007	Šmarje pri Jelšah	Fox	Yes (HM852161)
306	2007	Brežice	Fox	No
1001	2007	Metlika	Fox	No
275	2008	Brežice	Fox	Yes (HM852154)
332	2008	Brežice	Dog	Yes (HM852156)
339	2008	Mirna peč	Fox	Yes (HM852157)
348	2008	Majšperk	Badger	Yes (HM852159)
518	2008	Ilirska Bistrica	Fox	No
537 (vaccine induced)	2008	Šentjur	Fox	Yes
589	2008	Zavrč	Fox	Yes
1123	2008	Majšperk	Fox	No
1209	2008	Videm pri Ptuju	Badger	Yes (HM852160)
1868	2008	Slovenska Bistrica	Fox	Yes
2254	2008	Kanal na Soči	Fox	Yes
2441	2008	Videm pri Ptuju	Horse	Yes (HM852162)
2525	2008	Postojna	Fox	Yes
3212	2008	Metlika	Fox	Yes
244	2008	Ptuj	Fox	Yes
569	2008	Gorišnica	Fox	Yes
817	2008	Makole	Fox	Yes
511	2008	Brežice	Fox	Yes
127	2008	Šmarje pri Jelšah	Fox	Yes
303	2008	Rogatec	Fox	Yes
117	2008	Majšperk	Fox	Yes
177	2008	Majšperk	Fox	Yes
335	2008	Majšperk	Fox	Yes
347	2008	Majšperk	Fox	Yes
407	2008	Brežice	Fox	No
413	2008	Šmarje pri Jelšah	Fox	Yes
488	2008	Ptuj	Fox	No
532	2008	Šmarje pri Jelšah	Fox	No
543	2008	Cirkulane	Fox	Yes
1090	2008	Šmarje pri Jelšah	Fox	Yes
371	2009	Ilirska Bistrica	Cow	Yes (HM852167)
370	2009	Ilirska Bistrica	Fox	Yes
538	2009	Loški potok	Fox	Yes
839	2009	Rogaška Slatina	Fox	No
806	2009	Majšperk	Fox	Yes
846	2009	Videm	Fox	Yes
866	2009	Kozina	Fox	Yes
928	2009	Ilirska Bistrica	Fox	Yes
938	2009	Rogaška Slatina	Fox	Yes
940	2009	Rogaška Slatina	Fox	Yes
994	2009	Videm	Fox	Yes
1010	2009	Loški potok	Fox	Yes
1085	2009	Koper	Fox	Yes (HM852166)
1123	2009	Ilirska Bistrica	Fox	Yes
1220	2009	Ptuj	Fox	Yes
1223	2009	Makole	Fox	Yes
1229	2009	Loški potok	Fox	Yes
1262	2009	Loška dolina	Fox	Yes
1263	2009	Loška dolina	Fox	Yes
1282	2009	Metlika	Fox	Yes

1341	2009	Kozina	Fox	Yes
299	2009	Videm	Fox	Yes
2201	2009	Rogaška Slatina	Fox	Yes
2203	2009	Središče ob Dravi	Fox	Yes
2204	2009	Sveta Trojica	Fox	Yes
2315	2009	Ljutomer	Fox	Yes
2076	2009	Rogaška Slatina	Fox	Yes
2384	2009	Majšperk	Fox	Yes
2414	2009	Podsreda	Fox	Yes
2415	2009	Rogaška Slatina	Fox	Yes
2440	2009	Šmarje pri Jelšah	Fox	Yes
2660	2009	Majšperk	Fox	Yes
3000	2009	Slovenske Konjice	Fox	Yes
3501	2009	Poljčane	Fox	yes
3283	2009	Šmarje pri Jelšah	Fox	yes
52	2010	Poljčane	Fox	Yes (HM852163)
755	2010	Žetale	Cow	Yes (HM852158)
1083	2010	Cerknica	Fox	No
1084	2010	Cerknica	Fox	Yes
1327	2010	Rogatec	Fox	Yes
1466	2010	Slovenske Konjice	Fox	Yes
1710	2010	Ptuj	Fox	Yes
1938	2010	Makole	Fox	Yes
2153	2010	Rogaška Slatina	Fox	Yes
2156	2010	Šmarje pri Jelšah	Fox	Yes
3083	2010	Poljčane	Fox	No
4476	2010	Podlehnik	Fox	Yes
5533	2010	Rače	Fox	Yes
5761	2010	Ptuj	Fox	Yes
7288	2010	Rače	Fox	Yes
7448	2010	Podlehnik	Fox	Yes
467	2012	Cirkulane	Fox	Yes
3511 (vaccine induced)	2012	Tolmin	Fox	Yes (KC522613)
9945 (vaccine induced)	2014	Ljubljana	Marten	Yes
21082 (vaccine induced)	2018	Ljubljana	Fox	Yes

Table S2. The presentation of detected rabies positive samples in domestic and wild animal species; data collected between 1979 and 2019 (laboratory archive data from Virology Unit, NVI, Ljubljana, Slovenia).

Year	Dog	Cat	Cattle	Horse	Other domestic animals	Red fox	Eurasian badger	European pine marten	Roe deer	Other wildlife animals	Number of positive cases	Number of all animals tested	Incidence of positive cases
1979	1	2	1	0	0	102	1	0	3	0	110	1198	9,18
1980	3	9	5	0	0	807	12	1	24	5	866	3414	25,37
1981	16	35	12	0	0	1637	67	5	69	10	1851	7913	23,39
1982	2	7	4	0	0	540	19	1	11	3	587	3739	15,70
1983	3	6	0	0	0	403	9	3	6	1	431	2595	16,61
1884	1	10	1	0	0	492	12	0	12	1	529	2385	22,18
1985	1	2	0	0	0	182	2	2	1	0	190	1497	12,69
1986	1	1	0	0	0	116	1	0	0	0	119	1145	10,39
1987	2	4	0	0	0	315	3	1	2	0	327	1235	26,48
1988	13	12	4	1	3	981	14	15	19	5	1067	2963	36,01
1989	8	13	2	1	0	773	29	10	9	2	847	2181	38,84
1990	6	12	0	0	0	327	6	4	3	2	360	1249	28,82
1991	2	13	2	0	0	260	5	6	0	1	289	1185	24,39
1992	6	4	1	0	0	203	4	10	6	4	238	1365	17,44
1993	8	13	1	0	1	476	14	10	7	1	531	2019	26,30
1994	12	11	4	1	0	753	21	19	19	2	842	2632	31,99
1995	12	24	4	1	4	997	11	20	15	1	1089	3787	28,76
1996	11	17	1	0	0	208	1	5	4	0	247	2285	10,81
1997	1	6	1	0	0	18	2	2	2	0	32	1224	2,61
1998	0	0	0	0	0	14	0	0	0	0	14	1381	1,01
1999	0	1	0	0	0	5	0	0	0	0	6	1195	0,50

2000	2	2	0	0	0	104	3	0	0	3	114	1509	7,55
2001	7	3	0	0	0	117	2	3	3	0	135	2153	6,27
2002	0	0	0	0	0	14	1	0	0	0	15	1495	1,00
2003	0	0	0	0	0	8	0	0	0	0	8	993	0,81
2004	0	0	0	0	0	2	0	0	0	0	2	1612	0,12
2005	0	0	0	0	0	3	0	0	0	0	3	1603	0,19
2006	0	0	0	0	0	2	0	0	0	0	2	1896	0,11
2007	0	0	0	0	0	3	0	0	0	0	3	2075	0,14
2008	1	0	0	1	0	51	2	0	0	0	55	2618	2,10
2009	0	0	1	0	0	34	0	0	0	0	35	2808	1,25
2010	0	0	1	0	0	15	0	0	0	0	16	2590	0,62
2011	0	0	0	0	0	0	0	0	0	9	9	2229	0,40
2012	0	0	0	0	0	3	0	0	0	0	3	2161	0,14
2013	0	0	0	0	0	1	0	0	0	0	1	1918	0,05
2014	0	0	0	0	0	0	0	1	0	0	1	2085	0,05
2015	0	0	0	0	0	0	0	0	0	0	0	1985	0,00
2016	0	0	0	0	0	0	0	0	0	0	0	1739	0,00
2017	0	0	0	0	0	0	0	0	0	0	0	1771	0,00
2018	0	0	0	0	0	1	0	0	0	0	1	1266	0,08
2019	0	0	0	0	0	0	0	0	0	0	0	1378	0,00
**TOTAL**	**119**	**207**	**45**	**5**	**8**	**9967**	**241**	**118**	**215**	**50**	**10975**	**86471**	**12,69**
